# A machine learning ensemble approach for 5- and 10-year breast cancer invasive disease event classification

**DOI:** 10.1371/journal.pone.0274691

**Published:** 2022-09-19

**Authors:** Raffaella Massafra, Maria Colomba Comes, Samantha Bove, Vittorio Didonna, Sergio Diotaiuti, Francesco Giotta, Agnese Latorre, Daniele La Forgia, Annalisa Nardone, Domenico Pomarico, Cosmo Maurizio Ressa, Alessandro Rizzo, Pasquale Tamborra, Alfredo Zito, Vito Lorusso, Annarita Fanizzi

**Affiliations:** 1 I.R.C.C.S. Istituto Tumori “Giovanni Paolo II”, Bari, Italy; 2 Dipartimento di Fisica and MECENAS, Università di Bari, Bari, Italy; 3 INFN, Sezione di Bari, Bari, Italy; Vellore Institute of Technology: VIT University, INDIA

## Abstract

Designing targeted treatments for breast cancer patients after primary tumor removal is necessary to prevent the occurrence of invasive disease events (IDEs), such as recurrence, metastasis, contralateral and second tumors, over time. However, due to the molecular heterogeneity of this disease, predicting the outcome and efficacy of the adjuvant therapy is challenging. A novel ensemble machine learning classification approach was developed to address the task of producing prognostic predictions of the occurrence of breast cancer IDEs at both 5- and 10-years. The method is based on the concept of voting among multiple models to give a final prediction for each individual patient. Promising results were achieved on a cohort of 529 patients, whose data, related to primary breast cancer, were provided by Istituto Tumori “Giovanni Paolo II” in Bari, Italy. Our proposal greatly improves the performances returned by the baseline original model, i.e., without voting, finally reaching a median AUC value of 77.1% and 76.3% for the IDE prediction at 5-and 10-years, respectively. Finally, the proposed approach allows to promote more intelligible decisions and then a greater acceptability in clinical practice since it returns an explanation of the IDE prediction for each individual patient through the voting procedure.

## Introduction

Breast cancer is one of the main causes of death worldwide [1]. For this reason, the management of breast cancer patients has always been a topic of great interest within the scientific community. Surgery of various types followed by adjuvant therapies, such as chemotherapy, hormonotherapy, radiotherapy alone or in combination, represents the primary breast cancer treatment [2, 3]. Despite great progress has been made to improve cancer survival [4], the choice of which adjuvant therapy must be performed for preventing the occurrence of invasive disease events (IDEs) after the primary tumor, such as recurrence, metastasis, contralateral and second tumors, still remains challenging [5, 6]. Clinical experts make their decisions for each single patient following relevant guidelines [2], after having collected and evaluated a series of measurements of clinical and histological parameters. So far, several research works have demonstrated the central role of breast cancer subtypes on both tumor prognosis and therapy efficacy [7, 8]. The first level breast cancer subtype classification involves immunohistochemistry-based subtypes, namely, Luminal-like, HER2-positive and Triple Negative tumors [9]. According to the identified subtype, patients eligible for a specific adjuvant treatment following surgery can be selected, thus sparing some other patients from unnecessary and/or potentially toxic treatments. However, due to the molecular heterogeneity of this disease, predicting the outcome and the efficacy of the adjuvant therapy tailored for each individual patient is very hard. Tools based on molecular biomarkers, such as mRNA biomarkers or genes, have also developed [10], complementing traditional histopathology methods. However, they are expensive and not all centers are provided with laboratories performing these types of analyses. Within this emerging scenario, the need of predictive models which make a trade-off between reliable predictions of therapy results and cost-effectiveness is urgent. In recent years, due to great advancements in the field of artificial intelligence applied to biomedicine, the design and development of machine learning models to support clinical decision-making processes in breast cancer treatment, have been extensively investigated in the state-of-the-art [11, 12]. Their best potential consists of learning data models automatically without a prior hypothesized knowledge about variable interactions. Interdependence and complex nonlinear relationships among clinical data can be recognized [13]. Several studies based on machine learning have attempted to predict breast cancer recurrence/metastasis by solving a classification task [14, 15] or developing survival models [16]. The prediction of composite events, namely IDEs, can play a relevant role within the adjuvant clinical trial setting for breast cancer [17]. Indeed, it is known that chemotherapy treatments may cause second tumors [18]. However, there is a lack of research studies focusing on IDE prediction. The occurrence of IDEs in breast cancer patients has been recently predicted by a Chinese research team, only in terms of survival rates [19]. In this work, a novel ensemble machine learning approach has been developed to address the IDE prediction as a binary classification task: breast cancer patients for which IDEs have or have not occurred at both 5- and 10-year follow-ups were discerned. The respective classes of belonging were indicated as IDE and non-IDE. The model was based on voting among multiple models [20]. When a coherent prediction among the models was obtained for a given patient, the patient was finally classified into one of the two class, IDE or non-IDE, and a final classification score was assigned. Conversely, when no coherence was found among the multiple models, no answer was given for that patient. In this last case, the ensemble model did not express a decision for that patient. Data from a cohort of 529 patients referred to Istituto Tumori “Giovanni Paolo II” in Bari, Italy, were used to train and validate the proposed approach.

## Materials and methods

### Experimental data

This study was conducted according to the guidelines of the Declaration of Helsinki and approved by the Scientific Board of Istituto Tumori ‘Giovanni Paolo II’–Bari, Italy. The number of the Protocol approved by the Scientific Board of Istituto Tumori ‘Giovanni Paolo II’ (Bari, Italy) was 6629/21). Written consent was not required from subjects, as it is retrospective study and involves minimal risk. All data were fully anonymized before analysis.

The experimental data analyzed in the current paper refer to a cohort of patients who were registered to Istituto Tumori “Giovanni Paolo II” in Bari, Italy, for a first breast tumor diagnosis in the period 1995–2019. Patients who underwent a neoadjuvant chemotherapy for breast cancer and/or had metastasis *ab initio* and/or had carcinoma in situ and/or followed up less than 10 years, while no clinical events have occurred in the meantime, were excluded from this study. A total of 28 features related to the primary breast tumor and the performed therapy was collected for 529 patients, who respected the eligible criteria. The clinical information included age at diagnosis (abbr. age), presence of previous tumors (abbr. prev. tumor, values: Yes/No), tumor diameter (abbr. diameter, values: T1a, T1b, T1c, T2, T3, T4), multiplicity (abbr. multiple, values: Yes/No) histological subtype (abbr. hist. type, values: ductal, lobular, other), type of performed surgery (abbr. surgery, values: quadrantectomy/mastectomy), estrogen receptor (abbr. ER, % value), progesterone receptor (abbr. PgR, % value), cellular marker for proliferation (abbr. Ki67, % value), histological grade (abbr. grading, values according to Elston–Ellis scale: G1, G2, G3), human epidermal growth factor receptor-2 (abbr. HER2, value: Pos/Neg), HER2 score (abbr. HER2/neu+, values: 0,1,2,3), in situ component (abbr. in situ comp., values: absent, present but not typed, G1, G2, G3), lymphovascular invasion (abbr. LVI, values: absent, present but not typed, focal, extensive), lymph nodes status (abbr. status l., values: N0, N1, N2, N3), sentinel lymph nodes (abbr. sentinel l., values: Negative/Not done/ Positive), lymphadenectomy (abbr. dissection l., values: Yes/No), the number of eradicated lymph nodes (abbr. eradicated l.), the number of metastatic lymph nodes (abbr. metastatic l.). For those patients who were affected by bilateral or multiple tumors, the data referred to the greatest receptor expression were attributed. The data expressing the performed therapy were reported as in the following: chemotherapy (abbr. CT, values: Yes/No), hormone therapy (abbr. HT, values: Yes/No), trastuzumab (values: Yes/No), CT scheme (values: absent, Anthracycline (Anthra) + taxane, Anthra, taxane, CMF, other), HT scheme (values: absent, Tamoxifen (Tam), luteinizing hormone-releasing hormone analogues (LHRHa), Tam + LHRHa, aromatase inhibitor (AI), Tam + AI, LHRHa + AI, other), therapy combination (abbr. ther. comb, values: No, HT, CT, CT + HT, CT + trastuzumab, CT + HT + trastuzumab). Finally, information about CT duration expressed in months (abbr. CT months), time elapsed between the year of the first tumor diagnosis and surgery expressed in months (abbr. diag.—surg. months) and time elapsed between surgery and therapy initiation expressed in months (abbr. surg.- ther. months), were also collected. All the features’ distributions are summarized in Table 1. Absolute values as well as percentage values are reported for categorical variables, whereas median values and first (q_1_) and third (q_3_) quartiles are specified for continuous values. Missing data are indicated as NA. Before data analysis, missing data of a given patient were replaced with the corresponding values of the patient with complete data whose feature vector had minimum Euclidean distance from the feature vector of the given patient [21].

**Table 1:** Features’ distributions of the collected patients.

Features	Distribution	Features	Distribution
**Overall** (abs.; %)	529; 100%	N2 (abs.; %)	41; 7.8%
**Age**		N3 (abs.; %)	21; 4.0%
Median; [*q*_1_, *q*_3_]	51; [45, 60]	NA (abs.; %)	9; 1.7%
**Previous Tumors**		**Lymphadenectomy**	
Yes (abs.; %)	16 (3.0%)	No (abs.; %)	52; 9.8%
No (abs.; %)	513 (97.0%)	Yes (abs.; %)	466; 88.0%
**Tumor Diameter**		NA (abs.; %)	11; 2.2%
T1a (abs.; %)	19; 3.6%	**Sentinel Lymph Node**	
T1b (abs.; %)	45; 8.5%	Not Done (abs.; %)	438; 82.8%
T1c (abs.; %)	227; 42.9%	Positive (abs.; %)	33; 6.3%
T2 (abs.; %)	187; 35.4%	Negative (abs.; %)	52; 9.8%
T3 (abs.; %)	14; 2.6%	NA (abs.; %)	6; 1.1%
T4 (abs.; %)	21; 4.0%	**Eradicated lymph nodes**	
NA (abs.; %)	16; 3.0%	Median; [*q*_1_, *q*_3_]	19 [14,24]
**Multiplicity**		NA (abs.; %)	18; 3.7%
Yes (abs.; %)	108; 20.4%	**Metastatic lymph nodes**	
No (abs.; %)	419; 79.2%	Median; [*q*_1_, *q*_3_]	0 [0,2]
NA (abs.; %)	2; 0.4%	NA (abs.; %)	29; 6.0%
**Histologic Subtype**		**Chemotherapy**	
Ductal (abs.; %)	468; 88.5%	No (abs.; %)	148; 28.0%
Lobular (abs.; %)	43; 8.1%	Yes (abs.; %)	379; 71.6%
Other (abs.; %)	18; 3.4%	NA (abs.; %)	2; 0.4%
**Type of Surgery**		**Hormonotherapy**	
Quadrantectomy (abs.; %)	339; 64.0%	No (abs.; %)	157; 29.7%
Mastectomy (abs.; %)	190; 36.0%	Yes (abs.; %)	370; 69.9%
**ER**		NA (abs.; %)	2; 0.4%
Median; [*q*_1_, *q*_3_]	44 [0,80]	**Trastuzumab**	
NA (abs.; %)	5; 1.0%	No (abs.; %)	465; 87.9%
**PgR**		Yes (abs.; %)	63; 11.9%
Median; [*q*_1_, *q*_3_]	21 [0,70]	NA (abs.; %)	1; 0.2%
NA (abs.; %)	6; 1.2%	**CT scheme**	
**Ki67**		Absent (abs.; %)	148; 28.0%
Median; [*q*_1_, *q*_3_]	22 [10,40]	Anthra. + taxane (abs.; %)	82; 15.5%
NA (abs.; %)	11 22.6%	Anthra. (abs.; %)	123; 23.2%
**Grading**		taxane (abs.; %)	3; 0.5%
G1 (abs.; %)	48; 9.1%	CMF (abs.; %)	100; 18.9%
G2 (abs.; %)	231; 43.6%	other (abs.; %)	68; 12.9%
G3 (abs.; %)	229; 43.3%	NA (abs.; %)	5; 1.0%
NA (abs.; %)	21; 4.0%	**HT scheme**	
**HER2**		Absent (abs.; %)	157; 29.7%
Negative (abs.; %)	336; 63.5%	Tamoxifen (abs.; %)	29; 5.5%
Positive (abs.; %)	85; 16.1%	LHRHa (abs.; %)	4; 0.8%
NA (abs.; %)	108; 20.4%	Tamoxifen + LHRHa (abs.; %)	85; 16.1%
**HER2/neu+**		AI (abs.; %)	163; 30.8%
0 (abs.; %)	162; 30.7%	Tamoxifen + AI (abs.; %)	28; 5.3%
1 (abs.; %)	99; 18.7%	LHRHa + AI (abs.; %)	13; 2.5%
2 (abs.; %)	61; 11.5%	other (abs.; %)	44; 8.3%
3 (abs.; %)	71; 13.4%	NA (abs.; %)	6; 1.0%
NA (abs.; %)	136; 25.7%	**Therapy combination**	
**In Situ Component**		No (abs.; %)	4; 0.8%
Absent (abs.; %)	405; 76.6%	HT (abs.; %)	157; 29.7%
G1 (abs.; %)	22; 4.2%	CT (abs.; %)	116; 21.9%
G2 (abs.; %)	15; 2.8%	CT + HT (abs.; %)	187; 35.3%
G3 (abs.; %)	16; 3.0%	CT + trastuzumab (abs.; %)	24; 4.5%
present, not typed (abs.; %)	69; 13.0%	CT + HT + trastuzumab (abs.;%)	39; 7.4%
NA (abs.; %)	2; 0.4%	NA (abs.; %)	2; 0.4%
**Lymphovascular Invasion**		**CT months**	
Absent (abs.; %)	339; 64.1%	Median; [*q*_1_, *q*_3_]	3; [0, 5]
Focal (abs.; %)	101; 19.1%	NA (abs.; %)	14; 2.9%
Extensive (abs.; %)	29; 5.5%	**Diag.–surg. months**	
present, not typed (abs.; %)	60; 11.3%	Median; [*q*_1_, *q*_3_]	0 [0,0]
**Lymph Node Stage**		**Surg.–ther. months**	
N0 (abs.; %)	271; 51.2%	Median; [*q*_1_, *q*_3_]	1; [1, 1]
N1 (abs.; %)	187; 35.3% *(Continued)*	NA (abs.; %)	18; 3.7%

Absolute and percentage counts are reported in round brackets. For age, ER, PgR, Ki67, eradicated l. and metastatic l., CT months, diag.–surg. months, surg.–ther. months, the median value and first (q_1_) and third (q_3_) quartiles of the distribution are indicated in squared brackets. The number of missing values (NA) is also specified.

### Problem formulation

In this study, machine learning was used to predict the occurrence of invasive disease events at both 5- and 10-years in breast cancer patients who have shown a first infiltrating breast cancer. The term IDEs refer to composite clinical events, such as local and distant recurrence, contralateral invasive breast cancers, second primary tumors and death [17]. Two binary classification tasks to discriminate patients for which IDE have or have not occurred depending on whether the follow-up was fixed either at 5-years or at 10-years were formulated. The label *IDE class* indicated those patients for whom an event occurred within 5 or 10 years from the first tumor diagnosis date. A total of 142 patients of our database has shown an IDE within 5-years from the first tumor diagnosis, out of which 111 recurrence, 21 contralateral tumors and 10 second tumors, whereas 207 patients have shown an IDE within 10-years from the first tumor diagnosis, out of which 157 recurrence, 32 contralateral tumors and 18 second tumors. The label *non-IDE class* indicated the control cases defined as follow. For the 5-year IDE prediction model, patients with at least 10-year follow-up and without an event within that timeframe were considered as control cases (374 patients). Patients followed up less than 10 years, while no events were happened in the meantime, were not considered in the non-IDE class to avoid biases and noise, since they could develop an event shortly after 5-years. Under the same rationale, for the 10-year IDE prediction model, patients with at least 14-year follow up and without an event within that timeframe were identified as control cases (322 patients).

### Machine learning ensemble approach rationale

**Fig 1:**
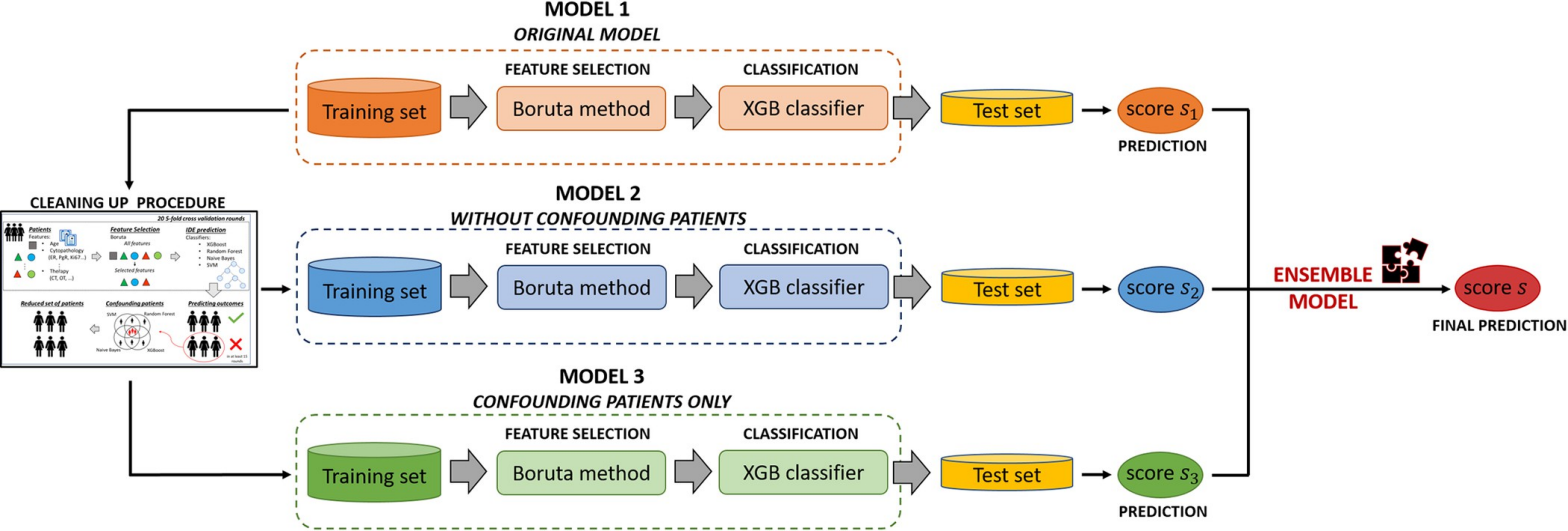
Workflow representing the diverse baseline models composing the proposed ensemble machine learning approach. Model 1 represents the original model which inputs the raw features referred to the training set. Model 2 is a model obtained after having applied a cleaning up procedure on the training set of Model 1: it does not involve the so-called confounding patients identified by means of the cleaning up procedure. Model 3 uses all the identified confounding patients as training set. The inner functioning of the three models consists of a feature selection by Boruta technique and, subsequently, of the training of an XGBoost (XGB) classifier, which is validated on an independent test set. The scores obtained by the three models are then combined according to specific rules, thus obtaining an ensemble model, which finally returns a prediction about the IDE occurrence. All the procedure is performed ten times independently and separately at 5-and 10-year follow-ups.

A predictive approach to give a prognostic prediction of IDE occurrence separately at 5- and 10-year follow-ups was designed. Its architecture consists of a machine learning-based ensemble approach which exploits the concept of voting among multiple models [20]. In other words, the proposed approach combines, according to specific rules described in S1 File, the predictions of three baseline models (Fig 1), named as Model 1, Model 2, Model 3. The approach was developed and evaluated on the entire dataset according to a 10-fold cross validation scheme in order to observe the performance over the entire dataset. In this way, training and test sets were created ten times independently by means of random sampling stratified with respect to the real class of belonging (IDE class/non-IDE class). Each test set was composed by the 10% of the total number of patients. In the following, the ten test sets are referred as *ten independent tests*. For each of the ten times, Model 1 took in input all the collected 28 features of the training set. In Fig 1 it is named as *original model* since it exploits the raw data. By starting from the training set of Model 1, a so-called *cleaning up procedure*, was performed thus obtaining another two models, Model 2 and Model 3. The details about the cleaning up procedure are reported in S1 File. The procedure is represented in S1 Fig. Basically, *cleaning up procedure* is based on four standard machine learning algorithms which classified the patients of the training set as IDE or non-IDE patients. All the patients marked as “misclassified” by all the classifiers were considered as *confounding samples*. By removing these patients from the training set, a model *without confounding patients*, Model 2, was then created. On the other hand, all the neglected patients were collected to define a new model, Model 3, *with confounding patients only*. The rationale under the concept of confounding patients is better clarified in the first sub-section of the Results section. Each of the three models was based on the same inner functioning procedure. First, a nested feature selection via Boruta technique has been applied on the training set through a 20 5-fold cross validation scheme rounds. Then, the selected features on the training set were used to train a machine learning classifier, eXtreme Gradient Boosting (XGBoost) [22], which returned a classification score for each patient of the test set (named as score *s1*,*s2*,*s3* for Model 1, Model 2, Model 3, respectively; see Fig 1). A patient of the test set was assigned by XGBoost classifier to the IDE-class if the predicted score exceeded a threshold, which was computed separately for each of the three models (and indicated as *th1*, *th2*, *th3*, respectively) as the ratio of the IDE-patients of the training set over the total amount of patients of the same set. As final step, an ensemble model was constructed by combining the classification scores obtained in correspondence of the three models according to the rules which are described in S1 File, and represented in S3 Fig. After the application of the rules, when a consensus among the three baseline models was obtained, the patient was finally classified into one of the two class, IDE or non-IDE, and a final classification score was assigned. Conversely, when no coherence among the three scores was judged by the rules, no answer was given for that patient. In this last case, the ensemble model did not express a decision for that patient. The rules at the basis of the ensemble model required the setting of 8 parameters that were computed from the distributions of scores returned by the three models on the ten training sets (see S1 File and S1 Table). A large set of trial solutions was examined by combining five trials for each of the parameters. A total of 5^8^ = 390625 parameter combinations across the ten independent tests were explored. To the aim, the solutions was drawn from a regular grid in the model space: this procedure was called *grid search*. A more detailed explanation of the grid search procedure in S1 File. All the experimental simulations were implemented using the R Software (v. 4.1.1, R Foundation for Statistical Computing, http://www.r-project.org/) and MATLAB R2019a (MathWorks, Inc., Natick, MA, USA) software. The models’ performance were evaluated in terms of the Area Under the receiver operating Curve (AUC) and, once the optimal threshold was identified by Youden’s index on ROC curve [23], standard metrics, such as accuracy, sensitivity and specificity were also computed:

Accuracy=(TP+TN)/(TP+TN+FP+FN) (1)

Sensitivity=TP/(TP+FN) (2)

Specificity=TN/(TN+FP) (3)

where TP and TN stand for True Positive (number of IDE cases correctly classified) and True Negative (number of non-IDE cases correctly classified), while FP (number of non-IDE cases identified as IDE cases) and FN (number of IDE cases identified as non-IDE cases are False Positive and False Negative ones, respectively. In the case of performance evaluation of the ensemble model, patients for which no prediction was given were not counted in any of the four categories (TP, TN, FP, FN), thus the number of “no answers” were computed [24, 25].

## Results

### Confounding patients

**Fig 2:**
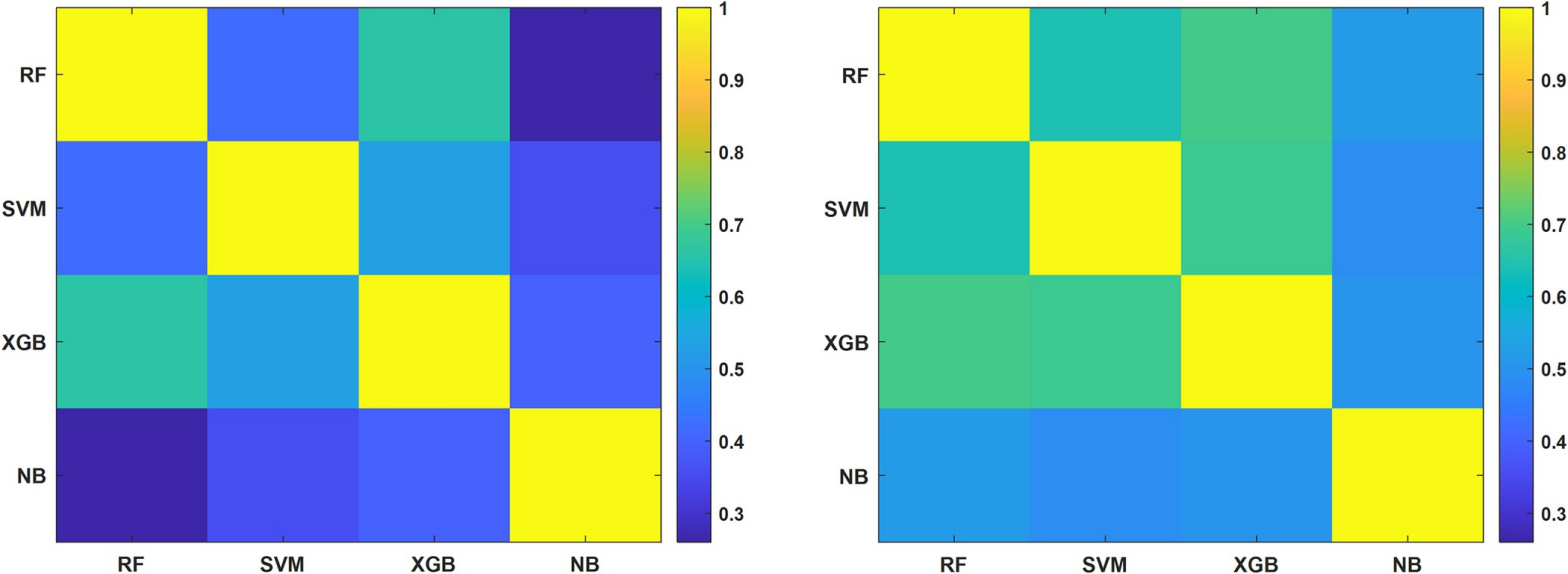
Consensus maps among each pair of the four classifiers, Random Forest (RF), Support Vector Machine (SVM), XGBoost (XGB) and Naïve Bayes (NB), (a) before and (b) after the cleaning up procedure applied for the 10-year IDE prediction. Consensus is measured by computing the Cohen’s kappa (*κ*) coefficient between each pair of classifiers on training sets and then averaged over 20 5-fold cross validation rounds.

Starting from the initial dataset, the classification tasks (at 5- and 10-years) were performed through Model 1, i.e., the original model taking in input the raw data. Model 2 was constructed from Model 1 by means of the implementation of the so-called cleaning up procedure based on four standard machine learning algorithms, such as XGB [22], Random Forest [26], Naïve Bayes [27] and SVM [28] (see S1 File). Confounding patients were identified as those patients marked as “misclassified” by all the classifiers. They were then excluded in the development of Model 2, whereas they were used to define Model 3. These patients were considered as confounding in model-independent way, since they appeared as indistinguishable with respect to the considered features for all the employed classifiers. Specifically, they presented similar clinical feature values to other patients of the database but belonged to opposite class. For this reason, it was not possible to define a unique model able to discern confounding patients to other patients. Fig 2 shows the consensus maps among the four classifiers computed in terms of Cohen’s kappa (*κ*) coefficient [29] between pairs of classification scores (e.g., XGBoost *vs* RF) before and after the cleaning up procedure applied for the 10-year IDE prediction (Fig 2A and 2B, respectively). The *κ* coefficient values range in the interval [-1;1]. The higher the *κ* coefficient, the greater the consensus is. Overall, the consensus among the classifiers increased after the cleaning up procedure (brighter values within the color map), thus meaning that by excluding confounding patients, the characteristics among the remaining patients are more homogeneous. The same behavior has been observed for the 5-year IDE prediction, but it is omitted to not burden the discussion.

### Performances of the baseline models composing the ensemble model

**Fig 3:**
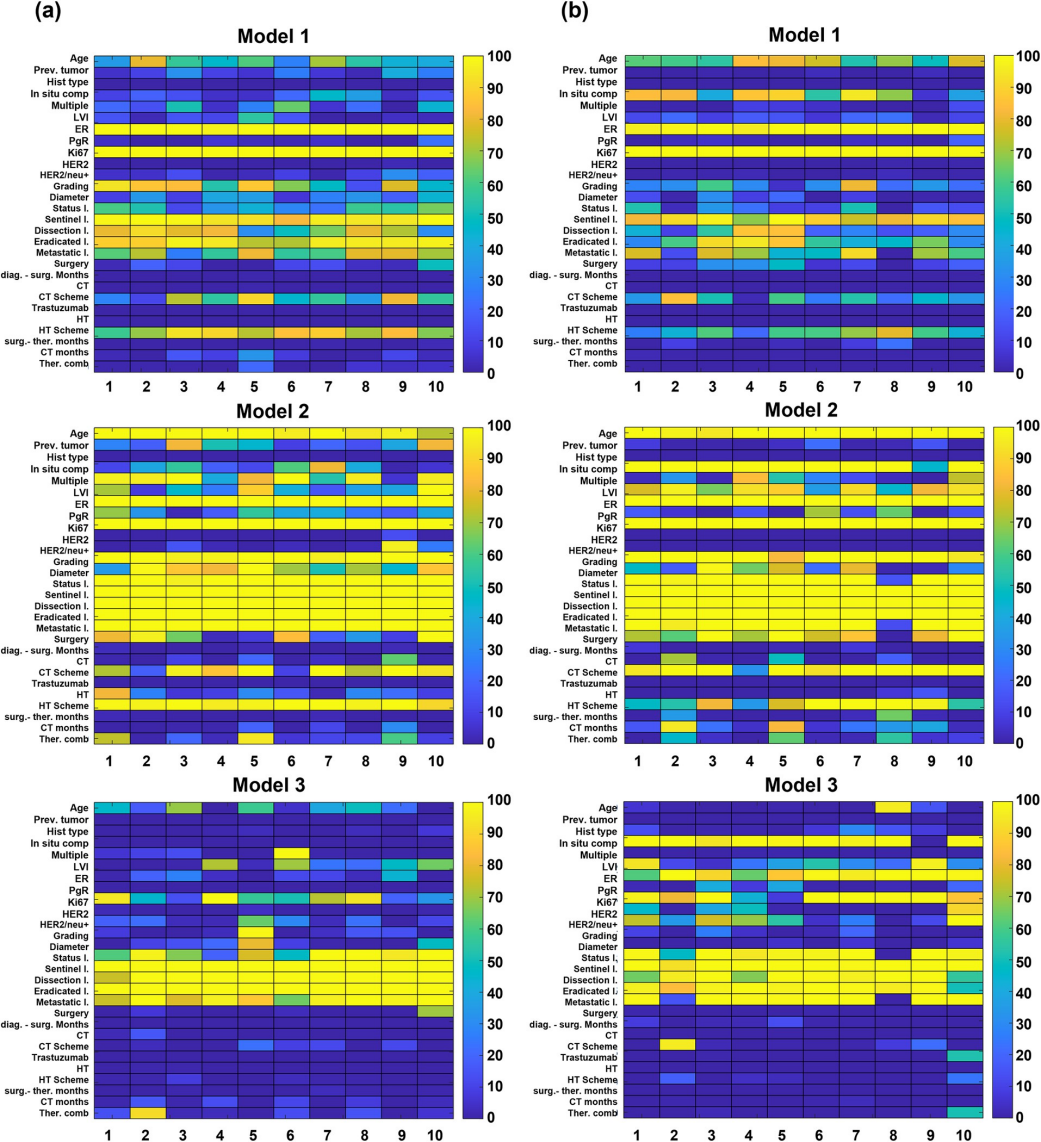
Maps representing the statistical frequency of features selected in nested cross-validation over the ten training sets for Model 1, Model 2 and Model 3. **(a)** at 5-year follow-up and **(b)** at 10-year follow-up. The statistical frequency is expressed in percentage values on the color bar: lower values correspond to dark colors; higher values correspond to brighter colors. The 28 collected features are disposed on the y-axis, whereas the training set identifiers from 1 to 10 are disposed on the x-axis.

The ensemble model proposed here, relies on the concept of voting among three baseline models. Here, the main results achieved by the three baseline models separately were discussed. For each of the three models, the statistical frequency of the features selected by means Boruta technique over each of the ten training sets though a 20 5-fold cross validation rounds for the 5-year follow-up and 10-year follow-up are depicted in Fig 3A and 3B, respectively. The most important features for Model 1 at both 5- and 10 follow-ups (with a percentage statistical frequency equal to 100% for all the ten training sets) are ER and Ki67, whose role is well known to be crucial in the decision of what therapy path to perform [8] (upper panels of Fig 3A and 3B). Other important features, selected especially for the 5-year follow up, are grading, the sentinel lymph node, the number of both eradicated and metastatic lymph nodes, lymphadenectomy, which, in the state-of-the-art, have been proven to be prognostic factors of recurrence [30, 31]. While grading reveals to be as an important feature for the 5-year follow up, in situ component, which has been recently recognized as a significant risk factor for intramammary recurrence [32], emerges as one of the most selected features for the 10-year follow-up. The number of selected features with a percentage statistical frequency of 100% increased for Model 2 at both 5- and 10-year follow-ups (central panels of Fig 3A and 3B). With respect to therapies, while HT scheme feature is mostly selected for the 5-year follow-up, the CT scheme is identified with a greater statistical frequency for the 10-year follow-up. The multiplicity feature emerges as important for the 5-year follow-up, whilst LVI and in situ component reach higher percentage statistical values for the 10-year follow-up. Finally, the most discriminative features for Model 3 at both the follow-ups (lower panels of Fig 3A and 3B) are represented by all the features related to lymph nodes. For the 10-year follow-up, other features, such as in situ component, ER and Ki67 reveal to be frequently selected. Table 2 highlighted the classification performances of the three baseline models, i.e., Model 1, Model 2, Model 3, over the ten training sets after having performed a nested feature selection by means of Boruta and trained a XGB classifier according to 20 5-fold cross-validation rounds scheme. The percentage median value as well as the percentage first and third quartile values of all the evaluation metrics were computed. Model 1 shows the lowest performances (a median AUC value of 68.1% and 68.0%, a median accuracy value of 66.8% and 64.3% for the 5-year follow-up and 10-year follow-up, respectively), whereas Model 2 as well as Model 3 achieved greater performances (a median AUC value of 94.8% and 83.8%, and a median accuracy value of 88.8% and 75.8% for the 5-year follow-up; a median AUC value of 94.4% and 89.9%, and a median accuracy value of 87.0% and 82.4% for the 10-year follow-up). Table 3 summarizes the percentage median, first and third quartile values of all the evaluation metrics achieved by all the three models on the ten independent tests. The AUC values reached by Model 2 decreased up to around 14%: a median AUC value of 70.5% and 70.7% were obtained for the 5-year and 10-year follow-up, respectively. As emerged from Table 3, Model 3 was not able to obtain satisfying results when tested on a set of patients involving not only confounding patients (the reached median AUC values are equal to 34.3% and 34.8% for the 5-year follow-up and 10-year follow-up, respectively). Conversely, Model 1 achieved stable performances when validated on the independent tests: median AUC values of 65.8% and 67.9% were obtained for the 5-year follow-up and 10-year follow-up, respectively.

**Table 2:** Summary of the performances for the 5- and 10-year follow-ups achieved by Model 1, Model 2, and Model 3 over the ten training sets after applying a 20 5-fold cross validation procedure.

Follow-up	Model	AUC (%)	Accuracy (%)	Sensitivity (%)	Specificity (%)
	Model 1	68.1 [66.8–69.4]	66.8 [65.9–68.1]	52.8 [50.8–55.0]	72.3 [71.1–73.6]
5 years	Model 2	94.8 [93.7–95.7]	88.8 [87.7–90.0]	81.9 [78.7–84.6]	91.2 [90.1–92.0]
	Model 3	83.8 [79.5–86.8]	75.8 [71.6–79.1]	77.1 [71.4–81.3]	73.7 [69.6–78.6]
10 years	Model 1	68.0 [66.7–69.6]	64.3 [62.9–65.3]	58.0 [55.9–60.1]	68.0 [66.3–69.4]
	Model 2	94.4 [93.9–95.5]	87.0 [85.6–88.2]	83.5 [81.5–85.2]	88.9 [87.4–90.3]
	Model 3	89.9 [86.0–92.3]	82.4 [78.5–85.1]	84.0 [79.6–87.6]	80.0 [73.5–84.4]

The performances are evaluated in percentage median values of AUC, accuracy, sensitivity, and specificity. The percentage first and third quartile values are also computed and reported in brackets.

**Table 3:** Summary of the performances for the 5- and 10-year follow-up achieved by Model 1, Model 2, and Model 3 over the ten independent test sets.

Follow-up	Model	AUC (%)	Accuracy (%)	Sensitivity (%)	Specificity (%)
	Model 1	65.8 [63.1–68.9]	65.1 [63.4–70.6]	49.2 [42.8–57.1]	70.7 [67.6–74.3]
5 years	Model 2	70.5 [67.7–73.4]	71.1 [67.3–74.5]	50.1 [42.9–63.7]	**77.3** [69.3–81.2]
	Model 3	34.3 [29.1–40.6]	35.9 [32.7–40.4]	35.5 [35,3–57.1]	31.9 [28.2–35.1]
	Ensemble Model	**77.1 [69.3–78.6]**	**75.7 [70.3–77.5]**	**64.0 [55.6–66.6]**	75.5 **[73.9–84.0]**
10 years	Model 1	67.9 [59.8–70.3]	63.2 [58.5–67.9]	62.2 [56.0–66.7]	62.1 [58.8–71.9]
	Model 2	70.7 [59.6–76.7]	66.0 [62.3–69.8]	51.3 [47.6–57.9]	75.8 [**64.7**–80.6]
	Model 3	34.8 [31.7–43.6]	41.5 [37.7–47.2]	50.0 [42.1–52.4]	38.5 [32.3–46.4]
	Ensemble Model	**76.3 [62.8–76.8]**	**71.3 [68.0–74.9]**	**66.0 [50.0–71.8]**	**81.9** [61.3–**87.5**]

The performances are evaluated in percentage median values of AUC, accuracy, sensitivity, and specificity. The percentage first and third quartile values are also computed and reported in brackets. For each metric, the best performances are highlighted in bold.

### Performances of the proposed machine learning ensemble approach

**Fig 4:**
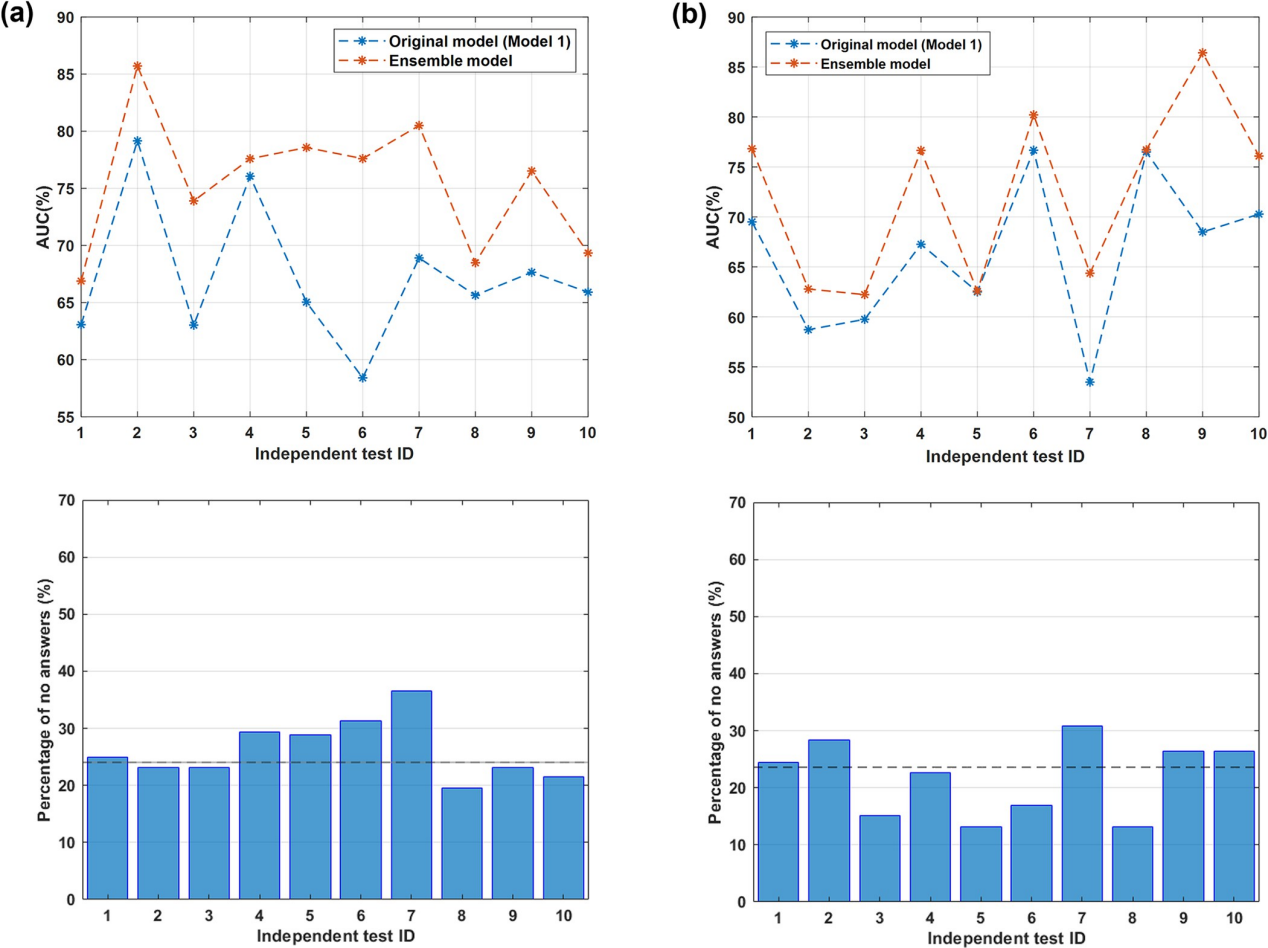
Comparison of the AUC values achieved by applying either the proposed ensemble model (orange lines) or the original model (blue lines) and the percentage number of no answers over each of the ten independent tests (a) at the 5-year follow-up and (b) at the 10-year follow-up.

Despite being stable between the training and the test sets, the results achieved by Model 1 do not make this model as being utilized in the actual clinical practice. Experimental results shown that a hidden pattern could be identified both by excluding confounding patients (Model 2) and by considering all confounding patients only (Model 3), but their performances show a gap when applied on the test sets. By merging the decisions of the three models, an ensemble approach that reached promising results was obtained, thus enabling its application in the actual clinical practice. Here, the performance achieved by the optimal ensemble model (in terms of AUC value) with a unique combination of parameters valid for all the ten independent test by imposing a percentage median value for the “no answers” given by the model over all the sets of patients as maximum 25% was presented. The percentage median values as well as the first and the third quartile values of all the evaluation metrics for both 5- and 10-year follow-ups are summarized in Table 3: a median AUC value of 77.1% and 76.3%, a median accuracy value of 75.5% and 71.3% were achieved for the 5-year follow-up and the 10-year follow-up, respectively. The model was optimized in terms of the AUC metric since it is the only metric not depending on the threshold identified by the computation of Youden’s index. Anyway, the ensemble model outperformed all the three baseline models with an overall improvement for all the other evaluation metrics. A direct comparison with the original model, i.e., Model 1, is also reported in the following. The upper panels of Fig 4A and 4B depict the AUC value achieved by the proposed ensemble model over all the ten independent tests separately (orange lines) alongside the AUC values obtained by Model 1 on the same sets (blue lines) for the 5-year follow-up and the 10-year follow-up, respectively. In the lower panels of Fig 4A and 4B, the percentage of the “no answers” returned by the ensemble model over all the independent test sets for the 5-year follow-up and the 10-year follow-up is shown, respectively. The median values of “no answers”, which are 23.4% and 23.6% for the 5-year follow-up and 10-year follow-up, respectively, are also reported as black dotted lines. An overall improvement of AUC values over all the independent test sets was obtained, especially for the 5-year follow-up. Here, a direct comparison of the ensemble model with Model 2 and Model 3 was neglected since they were derived from Model 1, which is also the most stable model with respect to the evaluation on the training and test sets.

## Discussion

An accurate prediction of breast cancer recurrence and analogous events could aid doctors in making better decisions about adjuvant treatment planning with an improvement in cost reduction and prevention of excessive treatment [33, 34]. Therefore, predicting the occurrence of recurrence in term of survival or classification has become a major issue in the current research on breast cancer. The 5-year follow-up is the most common benchmark in the breast cancer research field [16]. However, since breast cancer patients often experience events in a longer time and then longer-term therapy effect (e.g., the effect of hormone therapy) needs to be probed [35], also a 10-year follow-up is also frequently considered. PREDICT and Adjuvant! Online are two of the most popular tools at international level [36, 37], which are able to give prediction about the occurrence of recurrence probability for breast cancer patients in terms of survival, as they have been validated on diverse cohort of breast cancer patients in both United States and Western Europe. The subject of IDE prediction, despite being less investigated in the state of the art, is of great interest in the adjuvant clinical trial setting [17]. Treatment-related causes may play a role in the occurrence of second tumors or contralateral breast cancers [18, 38]. To date, results are inconclusive and, hence, the prediction of occurrence of composite events, i.e., IDEs, need to be investigated. The IDE prediction has been recently probed for the first time by Fu et al. [19]. They developed a 5-year survival model based on XGBoost which made use of patients’ characteristics related to demographics, diagnosis, pathology, and therapy. In this work, we wanted to make a contribution in the field of breast cancer IDE prediction. We developed a novel ensemble machine learning approach, based on the concept of voting among multiple models, which is able to predict in terms of classifications the occurrence of invasive disease events after the primary tumor, such as recurrence, metastasis, contralateral and second tumors at both 5- and 10-years follow-ups. The developed method has revealed to obtain promising performances on ten independent tests for both the follow-ups: a median AUC 77.1% and 76.3%, a median accuracy value of 75.5% and 71.3% were achieved for the 5-year follow-up and the 10-year follow-up, respectively. The method was also able to outperform the original predictive method, which took in input all the raw data of the entire dataset and returned a median AUC value of 68.1% and 68.0%, a median accuracy value of 66.8% and 64.3% for the 5-year follow-up and 10-year follow-up, respectively. A fundamental peculiarity of the proposed model is the ability to identify the so-called confounding patients and exploit them to define a consensus-based model. To the best of our knowledge, the proposed model is the first ensemble model within the field of IDE prediction, thus proposing an innovative angle from which probe and address the IDE prediction task. The concept of voting on which the model is based allow us to obtain better clarifications about the decision made by the classifier than standard machine-learning models, that, even if based on sophisticated mathematical underpinnings, usually share the common trait to fail in explaining in transparent and easy ways how a specific decision is achieved, thus hindering their applicability in clinical practice. The achieved promising results make this work as a first effort towards the implementation of a more intelligible method. However, at this step, the proposed model cannot be implemented in clinical practice yet since it required a validation on a wider cohort of patients, preferably including data collected across multiple centers. Moreover, this study has the limitation to have analyzed a heterogeneous sample population, since the period of the first tumor diagnosis was over 20 years (from 1995 to 2019). During this period, several pharmacological and treatment generations are being succeeded with a great impact on the predicted outcome. As example, the introduction of regimens containing anthracycline and taxane administered sequentially or in combination has resulted in a reduction of 16% of the risk of recurrence [39]. Randomized clinical studies within the adjuvant treatment framework, such as HERA, NSABP B-31, NCCTG N9831 and BCIRG 006, have also demonstrated how the addition of Trastuzumab to the typical therapy schemes has dramatically changed the natural history of HER2/neu+ breast cancer patients [40]. However, the developed model has learned to recognize even the effects of the first-generation drugs such as CMF, which, even if more rarely, are still used in clinical practice alongside new generation drugs. As future works, a wider cohort of patients will be used to evaluate the model generalizability and robustness alongside to the development of a tool that is able to distinguish the specific typology of the invasive disease event (recurrence, contralateral breast cancer and second tumor). The addition of radiomic features extracted by primary tumor diagnostic imaging exams, e.g., ultrasounds or mammograms, could be also investigated [41, 42].

## Conclusions

The current work presents a novel ensemble machine learning approach that, based on voting among three baseline models and grid search procedure, was able to predict the occurrence of 5- and 10-year invasive disease events in breast cancer patients. When a coherent prediction among the baseline models was obtained, the model returned a specific prediction for a given patient. Conversely, when no consensus among the baseline models was reached, the ensemble model remained unexpressed about that patient. The identification of confounding patients as well as the definition of an ensemble method, that returned a decision only when a consensus among the inner classifiers is reached can be considered as innovative aspects with respect to IDE prediction challenge. In the vein of the bursting concept of explainable artificial intelligence within the biomedical field, this ensemble model is able to return more intelligible choices as it is based on the concept of voting among multiple models. This aspect is particularly important for an easier clinical applicability of a decision support system that, just as the model proposed here, shows promising results. A greater interpretability of the results could lead clinicians to be more prone to adopt medical artificial intelligence methodologies in the actual clinical practice.

## Supporting information

S1 File Details about model design. (DOCX)

S1 Fig Overview of the cleaning up procedure. (TIF)

S2 Fig Examples of distributions of the scores associated with the corrected classification within the non-IDE and IDE classes (indicated as ok-non-IDE and IDE, respectively), and the wrong classifications (indicated as wrong). They were computed for the 10-year IDE prediction by means of the XGB classifier after implementing a 20 5-fold cross validation round scheme over one out of the ten training sets.
(TIF)

S3 Fig Workfolw of the rule process at the basis of the ensemble model. The scores *si*, *sj*, *sk* are the scores of Model 1, Model 2 and Model 3, with i,j,k ∈ {1,2,3}, the score *s* is the final prediction of the ensemble model.
(TIF)

S4 Fig Distributions of AUC values reached for the 5-year IDE prediction to varying each of the possible values that each of the eight parameters can assume for each of the ten independent tests separately. The word av. stands for average. The word q stands for quantile. The order of each q is also specified.
(TIF)

S5 Fig Distributions of AUC values reached for the 5-year IDE prediction to varying each of the possible values that each of the eight parameters can assume for each of the ten independent tests separately. The word av. stands for average. The word q stands for quantile. The order of each q is also specified.
(TIF)

S6 Fig Best performances reached by the ensemble model for the 5-year IDE prediction. (a) Comparison of the best AUC values achieved by applying the proposed ensemble model (orange line) with the AUC values reached the original model (blue line) over each of the ten independent tests (left panel), and percentage number of no answers over each of the ten independent tests (right panel). (b) Distribution of the eight parameters for which the grid search procedure was performed across the ten independent test sets.
(TIF)

S7 Fig Best performances reached by the ensemble model for the 5-year IDE prediction. (a) Comparison of the best AUC values achieved by applying the proposed ensemble model (orange line) with the AUC values reached the original model (blue line) over each of the ten independent tests (left panel), and percentage number of no answers over each of the ten independent tests (right panel). (b) Distribution of the eight parameters for which the grid search procedure was performed across the ten independent test sets.
(TIF)

S1 Table Summary of the parameters appearing in the rules of the ensemble model. For all the parameters, a brief description is reported in the second column. The range of variation represents all the possible values that a specific parameter could assume, whilst the step indicates the distance between two subsequent values within that range. The parameters th1, th2 and th3 do not require a range of variation since they were automatically computed on each of the ten training sets separately, as reported in the second column. Except for these three last parameters, the other eight parameters can assume five possible values and their optimal parameter combination under set conditions was identified by implementing the grid search procedure. Since these parameters were computed for the ten training sets separately, for each of them, a distribution was obtained, and the quantiles of certain order were computed. The extremes of the respective ranges were obtained by averaging the quantiles of specific order over the ten training sets. The number following the word quantile indicates the order of that quantile.
(DOCX)
