# Supplementary material for: A machine learning ensemble approach for 5- and 10-year breast cancer invasive disease event classification
Source: PLoS One. 2022 Sep 19;17(9):e0274691. doi: 10.1371/journal.pone.0274691 (PMC9484691; doi:10.1371/journal.pone.0274691)
Supplement: S1 File — (DOCX) [file pone.0274691.s001.docx]

**Details about model design**

**Cleaning up procedure**

The training sets of both Model 2 and Model 3 were obtained by applying the so-called cleaning up procedure to the training set of Model 1 (S1 Fig). This procedure allowed us to identify the confounding patients and was applied for the 5- and 10-year follow-ups separately by implementing a 20 5-fold cross validation rounds scheme. First, starting from all the 28 features collected for the training set of Model 1, a nested feature selection procedure via Boruta technique was applied to identify the most relevant features for the IDE prediction. These features were taken in input by four well-known classifiers, that are XGB [1], Random Forest [2], Naïve Bayes [3] and SVM [4], and an IDE prediction was obtained by each of the classifiers. For each classifier, the patients wrongly classified in at least 15 over 20 rounds were identified, where 15 over 20 corresponds to the third quartile upper bound. The wrongly classified patients in common at all the four classifiers were discarded and the remaining patients formed a reduced set of patients. The patients that were discarded after the application of the procedure were indicated as confounding patients.

In this analysis, the radial basis kernel function was employed for the SVM classifier. A configuration with 500 trees was adopted for the Random Forest classifier. To avoid overfitting, a small number of observations per tree leaf, such as 5, was fixed. XGBoost is a decision-tree strategy-based ensemble algorithm deriving from the gradient boosting framework, where each individual tree is a sort of “weak predictor”. The tuning of several incorporated parameters guarantees its robustness to overfitting. Among them, the booster parameters were related to the chosen booster, such as boosting learning rate (eta), number of boosted trees to fit (n_estimators), maximum tree depth for base learners (max_depth), minimum sum of instance weight needed in a child (min_child_weight), subsample ratio of columns (colsample_bytree), subsample ratio of the training instance (subsample). Similarly to the work of Fu et al. [5], the parameters were set as follows: eta = 0.1, n_estimators = 100, max_depth = 5, min_child_weight = 2, subsample = 0.9, col-sample_bytree = 1.0. Learning task parameters related to the learning scenario also need to be defined, such as the objective function (objective) and the evaluation metric (eval_metric) that measures the performance of the objective function as a return value. Other parameters were: objective = softprob, that returns predicted probability of each data point belonging to each class, and eval_metric = error, that is the binary classification error rate calculated as number of wrong cases over all cases. Moreover, XGB classifier was used to construct the baseline models at the basis of the proposed ensemble model, since it achieved the best performances on the training sets with respect to the other classifiers, in concordance with its superior performances reached in data mining competitions of the state-of-the-art with a drastically reduced computational time [1].

**Grid search at the basis of the proposed ensemble model**

The IDE predictions made by the ensemble model at 5- and 10-year follow-ups were obtained by combining the IDE predictions returned by the three baseline models, Model 1, Model 2, and Model 3, according to pre-determined rules. These rules depended on some parameters that needed to be set before the rules’ implementation. S1 Table reports all the parameters appearing in the rules (first column) with a brief description of how they were defined (second column). The parameters *th1*, *th2* and *th3* stand for the thresholds of the three models defined as the ratio of the IDE-patients of the training set over the total amount of patients of the same set. A patient was assigned to the IDE class by a model, if the related score for the IDE prediction exceeded the threshold related to that model. These thresholds were automatically computed over the ten training sets separately. For all the remaining eight parameters summarized in S1 Table, a range of variation (third column) as well as a step (fourth column) were fixed, where the range of variation represents all the possible values that a specific parameter could assume, whilst the step indicates the distance between two subsequent values within that range. These parameters were firstly computed for the ten training sets separately as described in the second column. In this way, for each of them, a distribution of their own values was obtained in correspondence of each training set, and the quantiles of certain order were computed. The extremes of the respective ranges were finally obtained by averaging the quantiles of specific orders over the ten training sets (in the following, the computation of the parameters *bound1_th1, bound2_th1, bound2_th1, bound2_th2* were explained). Considering the ranges of variation and steps reported in S1 Table, each of the eight parameters could take five possible values. To set the values of these eight parameters, a tuning procedure called *grid search*, which exhaustively considers all the parameter combinations, was implemented [6, 7]. A parameter combination, i.e., a combination for which all the parameters assume specific values within the respective fixed range, can be represented as a point of a grid. In the case reported in this work, the total number of points, namely, the number of all the parameter combinations was equal to 5^8^ = 390625. In correspondence of each parameter combination, a model can be built, and then its performance can be evaluated in accordance with the standard evaluation metrics. In this work, the goal of the application of this procedure was to identify the optimal parameter combination under set conditions across the ten independent tests. In this way, an optimal model with optimal performances under that fixed condition was obtained. The model was optimized with respect to the AUC metric, given that it is the only metric independent from the threshold defined by the Youden’s index. As example, in the sub-section “Performances of the proposed machine learning ensemble approach” of the main text, the performances achieved by the optimal model (in terms of AUC value) after imposing a percentage median value for the “no answers” given by the ensemble model over all the ten independent tests as maximum 25% were shown. Sub-section “Additional results” describes the results achieved in correspondence of other pre-determined conditions. Among the parameters to be optimized by the grid search procedure, there were *bound1_th1, bound2_th1, bound1_th2, bound2_th2.* They were described respectively as the lower and upper bounds of *wrong classifications* over the training set (i.e., each one of the ten training sets separately) for Model 1 (if the names end with *th1*) and Model 2 (if the names end with *th2*). In the following, the meaning of these four parameters with the support of S2 Fig was explained. First of all, all the IDE predictions made by XGB classifier after implementing a 20 5-fold cross validation round scheme over each training set for both Model 1 and Model 2 were considered. S2 Fig depicts an example for one out of the ten training sets for the IDE prediction at 10-year follow-up. The same rationale is still valid for the 5-year follow-up. According to the IDE prediction, it was possible to identify the distributions of the scores associated with the corrected classification within the non-IDE and IDE classes (indicated as ok-non-IDE and IDE in S2 Fig), and the *wrong classifications* (indicated as wrong in S2 Fig). The parameters *bound1_th1* and *bound1_th2*, according to their description, can assume the first quartile of the distribution of the wrong classifications of Model 1 and Model 2, respectively, as the maximum value (magenta lines in S2 Fig). The magenta arrow below the magenta line indicates that this parameter can assume values that are lesser than the quantile of order 0.25. Their values can reach values up to the quantile of order 0.05. The parameters *bound2_th1* and *bound2_th2*, according to their description, can assume the third quartile of the distribution of the wrong classifications of Model 1 and Model 2, respectively, as minimum value (green lines in S2 Fig). The green arrow above the green line indicates that this parameter can assume values that are greater than the quantile of order 0.75. They can reach values up to the quantile of order 0.95. Since all these four parameters can take different values across the ten training sets, the extremes of the respective ranges were defined as unique values for all the training set. Hence, after computing the quantiles of order 0.05 and 0.25 for the parameters *bound1_th1, bound2_th1,* and the quantiles of order 0.75 and 0.95 for the parameters *bound1_th2, bound2_th2*, respectively, these quantiles were averaged over all the ten training sets. The parameters *boundiff*, *par0, par1, par2*, represent other boundaries which appeared within the rules at the basis of the ensemble model and played a crucial role to determine the final decision of the model. Also, except for *boundiff*, the extreme values of the ranges related to the abovementioned parameters were averaged over the ten training sets.

**Rules at the basis of the proposed ensemble model**

After having described the parameters composing the proposed model, the rules that established the final prediction of the ensemble model were presented. Given a patient, a schematic workflow of all these rules at the basis of the ensemble model that led to a final IDE prediction for that patient is represented in S3 Fig. The red word START indicates the beginning of the rule process. As expressed in the legend, blue rhombs indicate conditions, i.e., if statements, while red rectangles enclose decisions obtained after accepting or rejecting specific conditions. The scores returned by Model 1, Model 2 and Model 3 are indicated as *s1, s2, s3,* respectively, whereas the final score is represented by *s*. These rules were designed in order to give more weight to Model 2 to the final decision with respect to Model 1 and Model 3, since Model 2 reached the best performances over the training sets (see results in sub-section “Performances of the proposed machine learning ensemble approach” of the main text, Table 3). Despite S3 Fig is explanatory of the followed rule process, some rules need to be clarified. The rule at the beginning of the process was related to a decision over the score *s2* with respect to the non-IDE class by hypothesizing that Model 2 was able to give stable classifications with respect to that class. Indeed, if the statement was accepted, the final score *s* assumed the value of the score *s2.* This can be justified by the behavior of Model 2 in the right panel of S2 Fig, namely, the distribution of the corrected classification for the non-IDE class made by Model 2 shows a fewer variability than those related to the corrected classifications for the IDE class. In other words, Model 2 was able to produce more stable scores for the non-IDE class than for the IDE-class. It is true that S2 Fig represents an example of corrected and wrong classifications for one out of the ten training sets in the case of IDE prediction at 10-years follow-up. Anyway, the same behavior it was observed for all the ten training sets in the case of IDE predictions at both 5-and 10-year follow-ups. The initial hypothesis was that this behavior could be extended to the ten test sets for the predictions at both follow-ups and then this first rule was designed. The second rule, instead, assigned a final score equal to the score *s2* only when Model 1 and Model 2 assigned the same label to a patient (IDE class and non-IDE class) and, at the same time, Model 2 returned a stronger prediction for that class. A prediction by Model 2 can be evaluated as stronger when the assigned score was lower for the non-IDE class and when was greater for the IDE-class than those assigned by Model 1. Then, the agreement among the scores *s1, s2, s3* was computed and only if the agreement was judged as strong (last four decisions in S3 Fig), a decision was finally assigned for the ensemble model and a score *s* was computed. With respect to the third statement, if respected, the maximum or the minimum of the scores *s1, s2, s3* were computed depending on whether the assigned class was the IDE class (computation of the maximum score), or the non-IDE class (computation of the minimum). Therefore, the computation of the quantity |1-mean(*si*,*sj*)-*sk*|, where two scores *si*, *sj* agree, while the third score *sk* is in disagreement, with i,j,k $\in$ {1,2,3}, need to be clarified. The agreement between the two scores indicates that the models, to which *si* and *sj* are referred, classified a patient in the same class (IDE or non-IDE), whereas the disagreement of *sk* with respect to *si* and *sj* means that the model, to which *sk* is referred to, classified the same patient in the opposite class assigned by the first two models. Here, an explicative example is reported. Let us assume that i=1, j= 2 and k = 3, and that Model 1 and Model 2 classify a given patient in the non-IDE class with scores *s1* = 0.10, *s2* = 0.15, and then a the mean score between s1 and s2 is mean(s1,s2)=0.125, whereas Model 3 classify the same patient in the IDE class with *s3* = 0.90. To make the scores referred to opposite classes as comparable among them, the complement of mean(*s1,s2*), i.e., 1-mean(*s1,s2*) = 0.875, which, in this form, correspond to the score referred to the IDE class, was firstly computed. In details, the scores *s1*, *s2* referred were converted to the non-IDE class to a score referred to the IDE-class: the new obtained score is comparable with *s3*, since they refer to the same class. As final result, it was possible to assert if 1-mean(*s1,s2*) is greater or lesser than *s3*. In the example that is being explored, *s3* is greater than 0.875 and, since the second last condition of the rule process is not respected, the final prediction correspond to assign that patient to the IDE-class, with *s3* = max (*s3*, 1-mean(*s1,s2*)). In this case, the scores are converted to scores related to the IDE-class and then the maximum is computed. With the same rationale exposed here, the minimum among scores was computed when all the scores were converted to scores related to the non-IDE class.

**Additional results**

To understand the role of the values taken by the parameters, for which the grid search was performed, on the final performances expressed in terms of AUC value, S4 and S5 Figs represent the distribution of AUC values at varying the values assumed by each parameter over each of the ten independent tests, when the IDE prediction is performed at 5-year follow up and 10-year follow up, respectively.

In the case of both IDE prediction at 5-and 10-year follow-ups, it can be observed how the most variable performances (in terms of AUC values) are achieved at varying the values of *bound1_th2* parameter: this parameter appears crucial to the final performances achieved, and it can be justified by the fact that some “no answers” taken by the ensemble model can depend on the values taken by this parameter (S3 Fig). Not less relevant, Model 2 appeared more stable in assigning patients to the non-IDE class (S2 Fig) and the values assumed by *bound1_th2* could influence such a stability. Sub-section “Performances of the proposed machine learning ensemble approach” of the main text shows the performance achieved by the optimal ensemble model (in terms of AUC value) with a unique combination of parameters valid for all the ten independent tests by imposing the following condition: the percentage median value for the “no answers” given by the model over all the sets of patients could be as maximum 25%. A unique combination of parameters satisfying this condition for the 5-year follow-up and 10-year follow-up separately was found. The parameters of the ensemble model for the 5-year IDE prediction took the following values: *bound1_th1* = average quantile 0.20, *bound2_th1* = average quantile 0.80, *bound1_th2* = average quantile 0.05, *bound2_th2* = average quantile 0.75, *par0* = average quantile 0.55, *par1* = average quantile 0.35, *par2* = average quantile 0.35, *bounddiff* = 0.05. The parameters of the ensemble model for the 10-year IDE prediction took the following values: *bound1_th1* = average quantile 0.15, *bound2_th1* = average quantile 0.80, *bound1_th2* = average quantile 0.15, *bound2_th2* = average quantile 0.90, *par0* = average quantile 0.40, *par1* = average quantile 0.35, *par2* = average quantile 0.35, *bounddiff* = 0.02. As additional results, by setting the condition to obtain the best performances in terms of AUC value for each of the ten independent tests separately, a diverse combination of parameters was identified for each of the ten independent test sets, when the IDE-prediction was performed at 5- and 10-year follow-ups. The top-left panels of S6a and S7a Figs depict the best AUC values achieved over the ten test sets separately for the IDE-prediction at 5-and 10-year follow up, respectively. The top-right panels of S6a and S7a Figs show the number of “no answers” for each of the ten test sets. Since the optimal combination of parameter was not unique in this case, S6b and S7b Figs represent how the eight parameters are distributed across the ten independent test sets. For what concern the 5-year IDE prediction, all the parameter distributions show a peak, except for the parameters *bound2_th1* and *bound2_th2* that, instead, are quite uniformly distributed or do not show a preferred choice for the values assumed by the parameter. In the case of 10-year IDE prediction, *bound2_th1* and *boundiff* are the only parameters that do not show a peak*.* Finally, with respect to the results shown in sub-section “Performances of the proposed machine learning ensemble approach” of the main text, the performances are greater (median AUC value of 78.2% and 78.9% for the IDE prediction at 5-year follow-up and 10-year follow-up, respectively) at the expense of a higher number of “no answers” (median “no answers” of 34.9% and 36.8% for the IDE prediction at 5-year follow-up and 10-year follow-up, respectively). Moreover, the optimal combination of parameters varies from test set to test set. To apply the proposed ensemble model in the actual clinical practice, a trade-off between the number of “no answers” and good performances must be considered. Not less, relevant, the generalizability of the model, corresponding, in this case, to the having found a unique optimal combination of parameters for all the test sets, should be guaranteed. Under these considerations, it was preferred to present as main results those explained in sub-section “Performances of the proposed machine learning ensemble approach” of the main text, rather than those discussed here.

**References**

1. Chen T, Guestrin C (2016) XGBoost : A Scalable Tree Boosting System. 785–794

2. Cutler A, Cutler DR (2011) Random Forests. https://doi.org/10.1007/978-1-4419-9326-7

3. Webb GI (2019) Naïve Bayes. https://doi.org/10.1007/978-1-4899-7502-7

4. Cyran KA, Kawulok J, Kawulok M, et al (2013) Support Vector Machines in Biomedical and Biometrical Applications Support Vector Machines in Biomedical and Biometrical Applications

5. Fu B, Liu P, Lin J, et al (2019) Predicting Invasive Disease-Free Survival for Early Stage Breast Cancer Patients Using Follow-Up Clinical Data. IEEE Trans Biomed Eng 66:2053–2064. https://doi.org/10.1109/TBME.2018.2882867

6. Michie D, Speigelhalter DJ, Taylor CC (1994) Machine Learning, Neural And Statistical Classification. 1

7. Feurer M, Hutter F (2019) Hyperparameter Optimization
