# Supplementary figures and images for: A machine learning ensemble approach for 5- and 10-year breast cancer invasive disease event classification
Source: PLoS One. 2022 Sep 19;17(9):e0274691. doi: 10.1371/journal.pone.0274691 (PMC9484691; doi:10.1371/journal.pone.0274691)

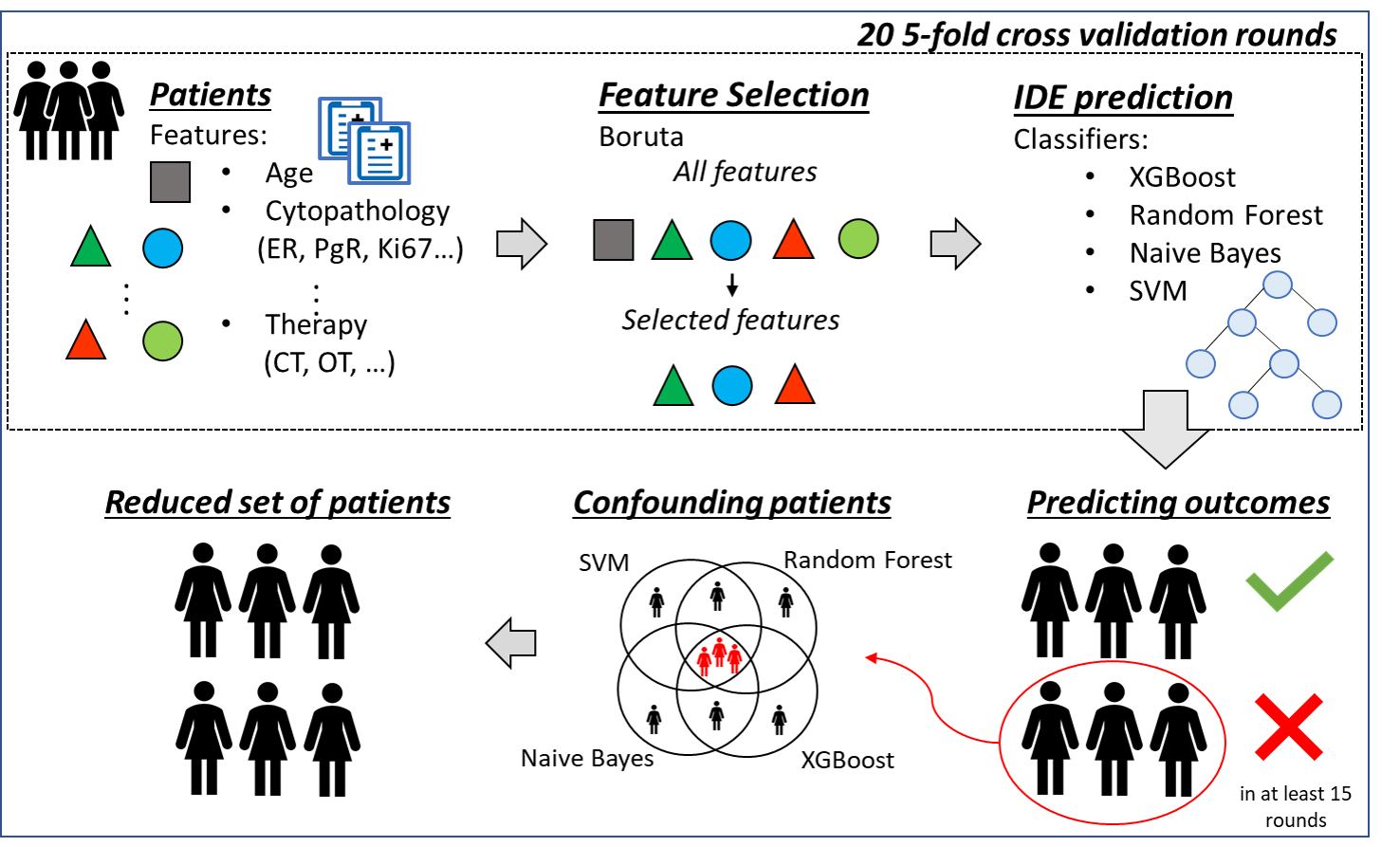

Supplement: S1 Fig — (TIF) [file pone.0274691.s002.tif]

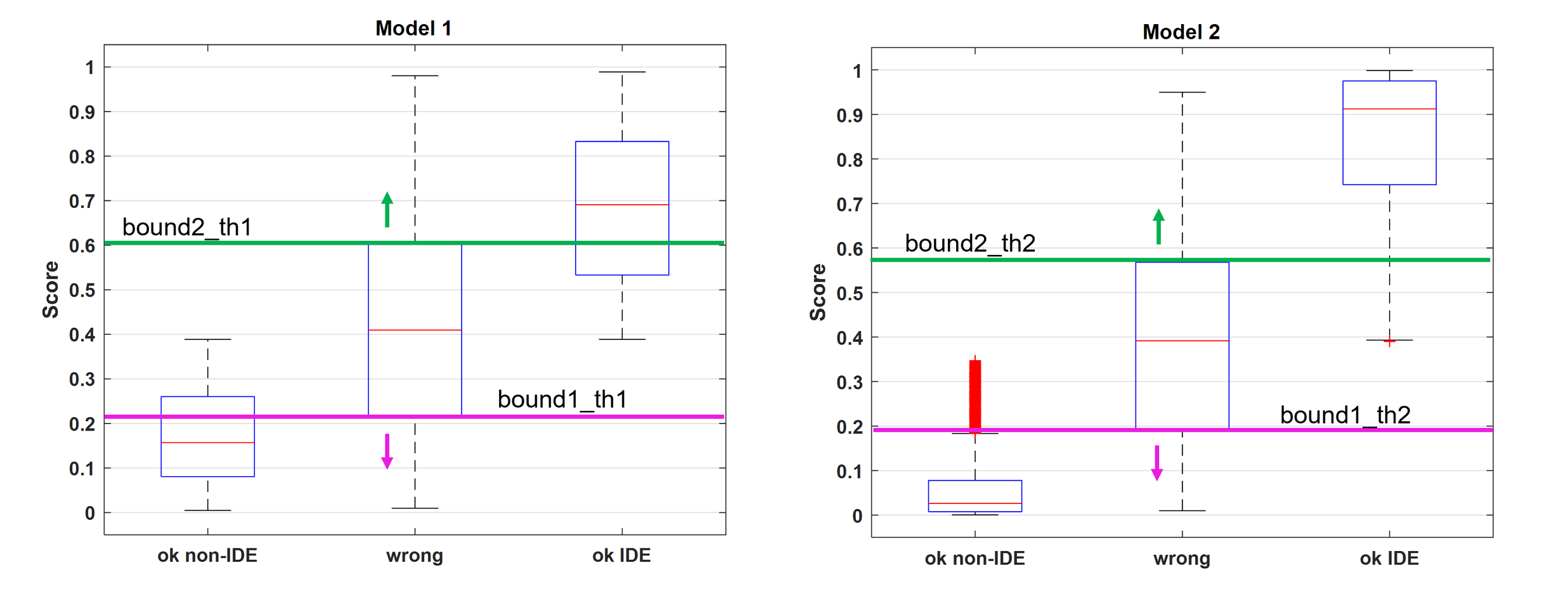

Supplement: S2 Fig — They were computed for the 10-year IDE prediction by means of the XGB classifier after implementing a 20 5-fold cross validation round scheme over one out of the ten training sets. (TIF) [file pone.0274691.s003.tif]

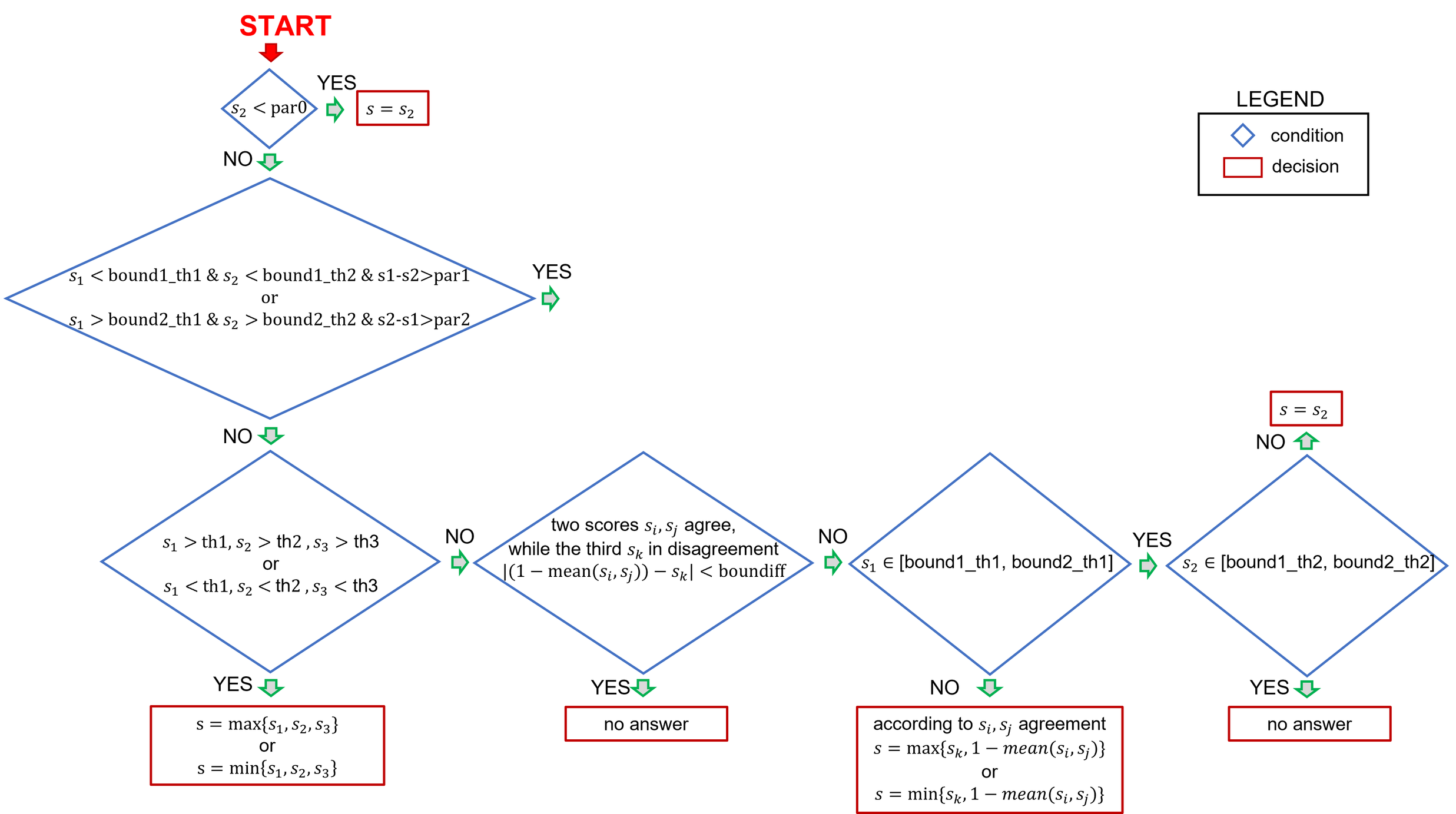

Supplement: S3 Fig — The scores si, sj, sk are the scores of Model 1, Model 2 and Model 3, with i,j,k ∈ {1,2,3}, the score s is the final prediction of the ensemble model. (TIF) [file pone.0274691.s004.tif]

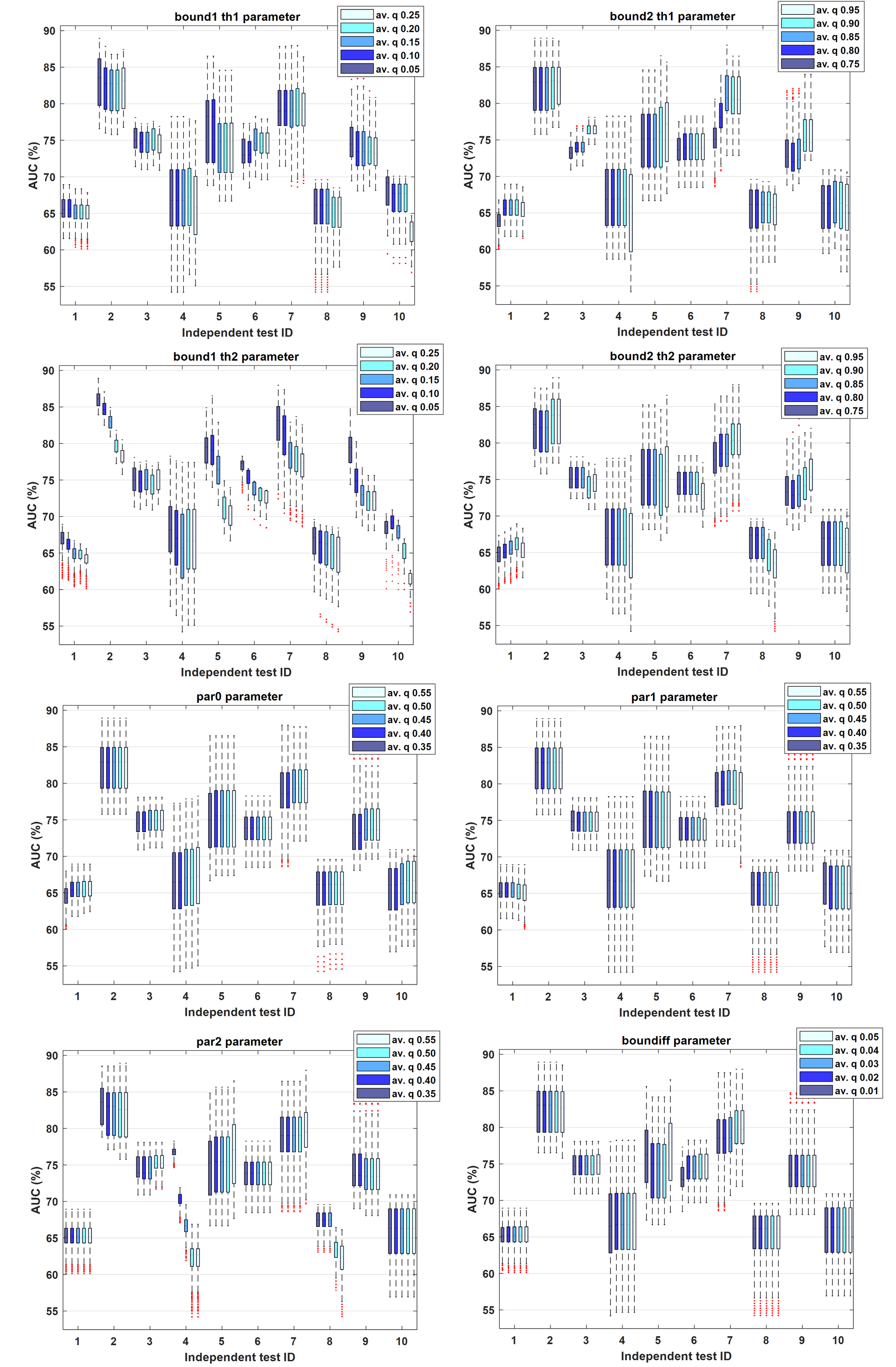

Supplement: S4 Fig — The word av. stands for average. The word q stands for quantile. The order of each q is also specified. (TIF) [file pone.0274691.s005.tif]

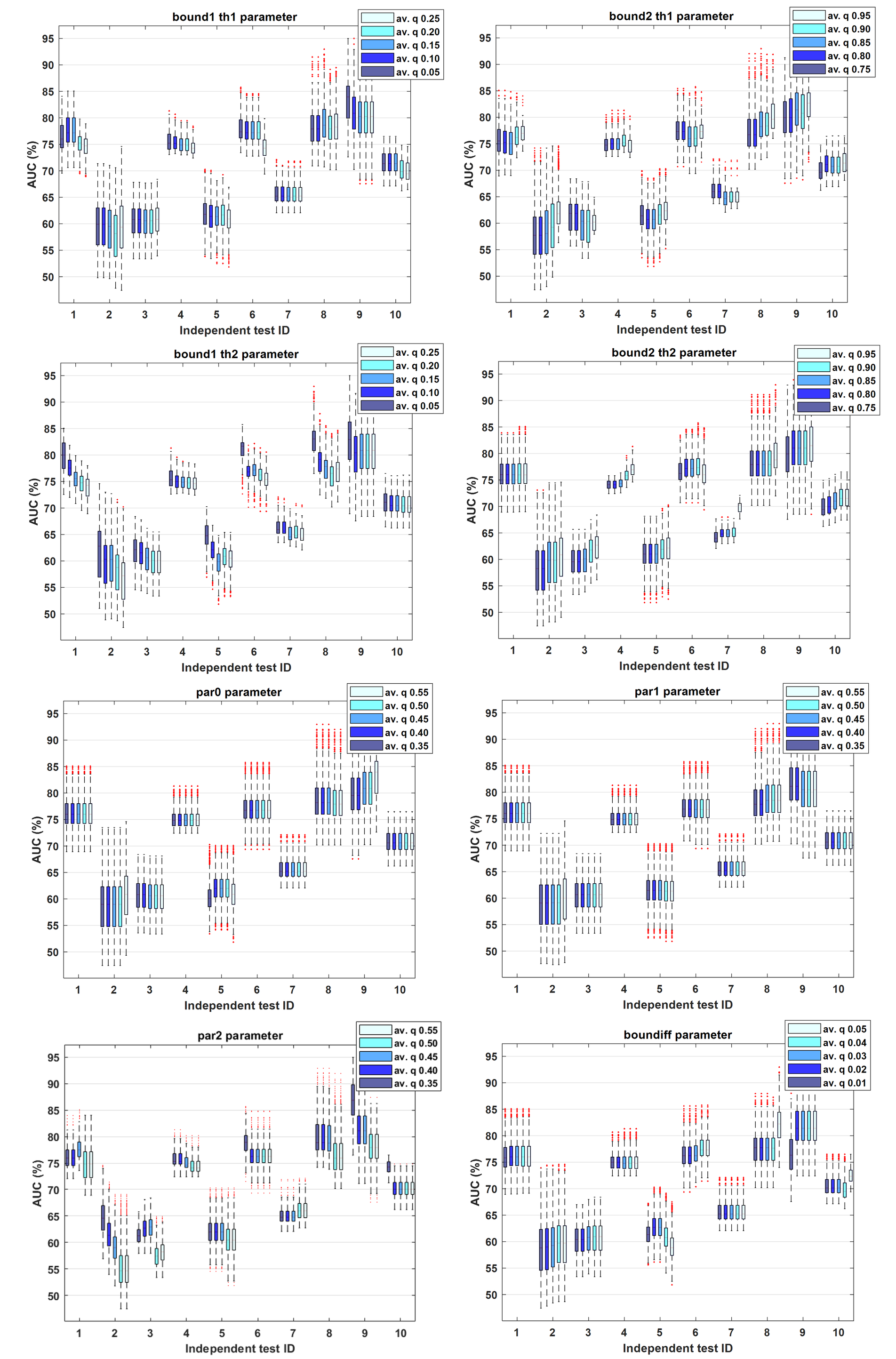

Supplement: S5 Fig — The word av. stands for average. The word q stands for quantile. The order of each q is also specified. (TIF) [file pone.0274691.s006.tif]

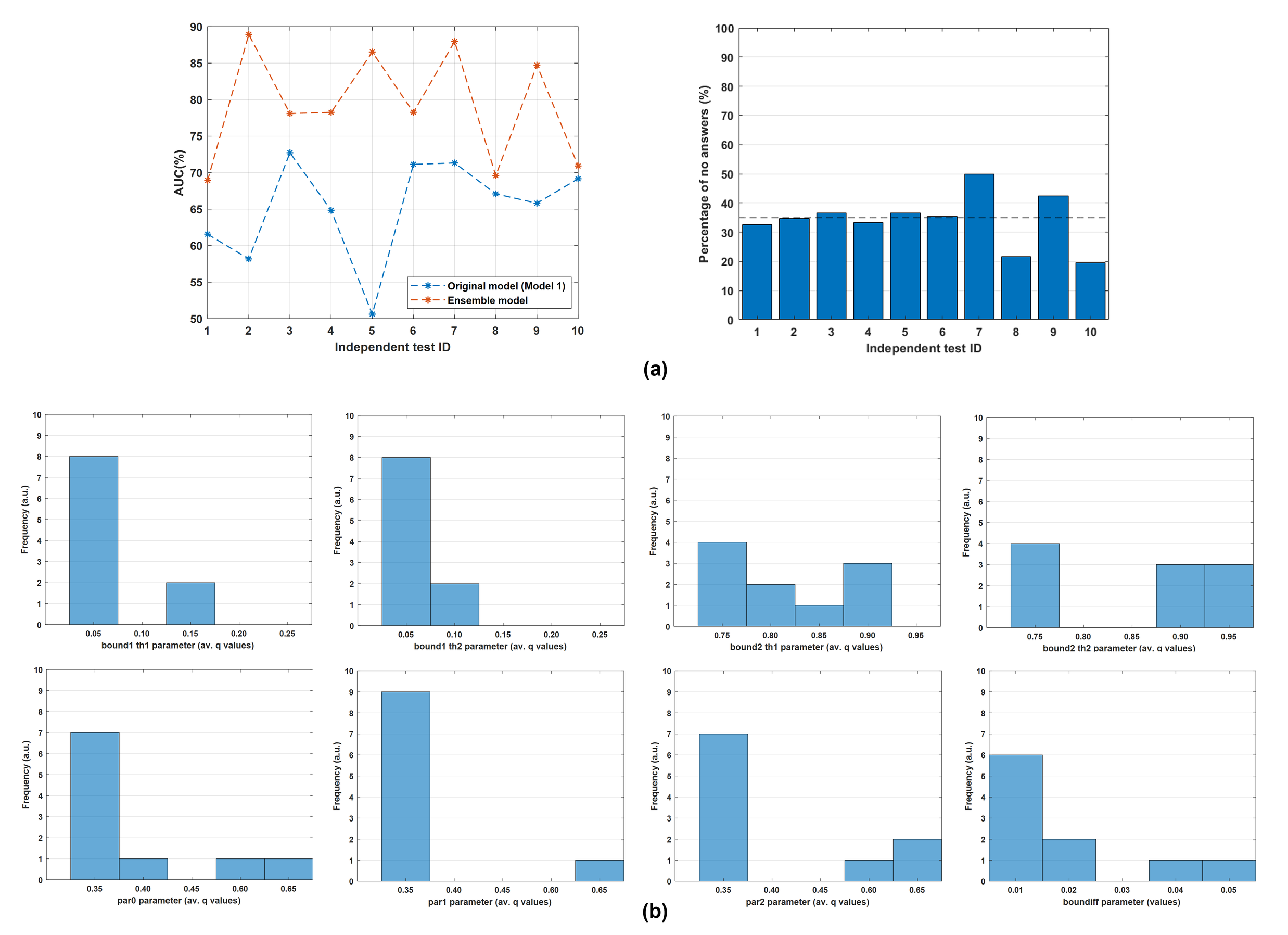

Supplement: S6 Fig — (a) Comparison of the best AUC values achieved by applying the proposed ensemble model (orange line) with the AUC values reached the original model (blue line) over each of the ten independent tests (left panel), and percentage number of no answers over each of the ten independent tests (right panel). (b) Distribution of the eight parameters for which the grid search procedure was performed across the ten independent test sets. (TIF) [file pone.0274691.s007.tif]

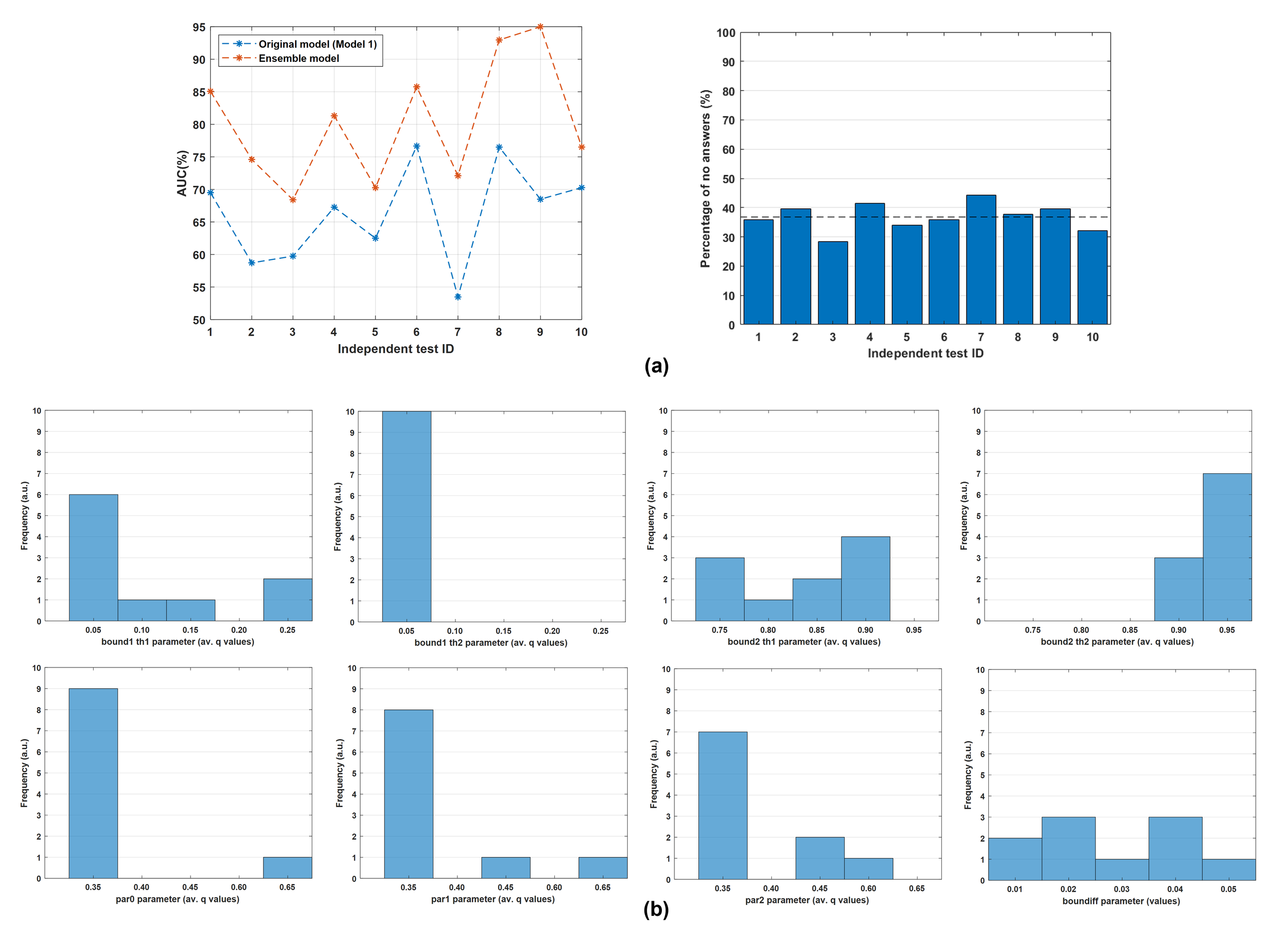

Supplement: S7 Fig — (a) Comparison of the best AUC values achieved by applying the proposed ensemble model (orange line) with the AUC values reached the original model (blue line) over each of the ten independent tests (left panel), and percentage number of no answers over each of the ten independent tests (right panel). (b) Distribution of the eight parameters for which the grid search procedure was performed across the ten independent test sets. (TIF) [file pone.0274691.s008.tif]
