# Supplementary material for: A machine learning ensemble approach for 5- and 10-year breast cancer invasive disease event classification
Source: PLoS One. 2022 Sep 19;17(9):e0274691. doi: 10.1371/journal.pone.0274691 (PMC9484691; doi:10.1371/journal.pone.0274691)
Supplement: S1 Table — For all the parameters, a brief description is reported in the second column. The range of variation represents all the possible values that a specific parameter could assume, whilst the step indicates the distance between two subsequent values within that range. The parameters th1, th2 and th3 do not require a range of variation since they were automatically computed on each of the ten training sets separately, as reported in the second column. Except for these three last parameters, the other eight parameters can assume five possible values and their optimal parameter combination under set conditions was identified by implementing the grid search procedure. Since these parameters were computed for the ten training sets separately, for each of them, a distribution was obtained, and the quantiles of certain order were computed. The extremes of the respective ranges were obtained by averaging the quantiles of specific order over the ten training sets. The number following the word quantile indicates the order of that quantile. (DOCX) [file pone.0274691.s009.docx]

| Parameter | Description | Range | Step |
| --- | --- | --- | --- |
| th1 | Threshold Model 1:  IDE/total DB on training set | - | - |
| th2 | Threshold Model 2:  IDE/total DB on training set | - | - |
| th3 | Threshold Model 3:  IDE/total DB on training set | - | - |
| bound1_th1 | Lower bound of wrong classifications for Model 1 on the training set | [average quantile 0.05; average quantile 0.25] | 0.05 |
| bound2_th1 | Upper bound of wrong classifications for Model 1 on the training set | [average quantile 0.75; average quantile 0.95] | 0.05 |
| bound1_th2 | Lower bound of wrong classifications for Model 2 on the training set | [average quantile 0.05; average quantile 0.25] | 0.05 |
| bound2_th2 | Upper bound of wrong classifications for Model 2 on the training set | [average quantile 0.75; average quantile 0.95] | 0.05 |
| bounddiff | Proximity bound of the score pair obtained by Model 1 and Model 2 | [0.01;0.05] | 0.01 |
| Par0 | Boundary for the distribution of the score obtained by Model 2 below th2 | [average quantile 0.35; average quantile 0.55] | 0.05 |
| Par1 | Difference boundary between the scores obtained by Model 1 and Model 2 for the class non-IDE (measure the score reduction by passing from Model 1 to Model 2) | [average quantile 0.35; average quantile 0.55] | 0.05 |
| par2 | Difference boundary between the scores obtained by Model 1 and Model 2 for the class IDE (measure the score increase by passing from Model 1 to Model 2) | [average quantile 0.35; average quantile 0.55] | 0.05 |
